# Mastering Complexity: Towards Bottom-up Construction of Multifunctional Eukaryotic Synthetic Cells

**DOI:** 10.1016/j.tibtech.2018.03.008

**Published:** 2018-09

**Authors:** Kerstin Göpfrich, Ilia Platzman, Joachim P. Spatz

**Affiliations:** 1 Max Planck Institute for Medical Research, Department of Cellular Biophysics, Jahnstraße 29, D 69120, Heidelberg, Germany; 2 Department of Biophysical Chemistry, University of Heidelberg, Im Neuenheimer Feld 253, D 69120 Heidelberg, Germany

**Keywords:** bottom-up synthetic biology, synthetic cell, droplet-based microfluidics, compartment, giant unilamellar vesicle, DNA nanotechnology

## Abstract

With the ultimate aim to construct a living cell, bottom-up synthetic biology strives to reconstitute cellular phenomena *in vitro* – disentangled from the complex environment of a cell. Recent work towards this ambitious goal has provided new insights into the mechanisms governing life. With the fast-growing library of functional modules for synthetic cells, their classification and integration become increasingly important. We discuss strategies to reverse-engineer and recombine functional parts for synthetic eukaryotes, mimicking the characteristics of nature’s own prototype. Particularly, we focus on large outer compartments, complex endomembrane systems with organelles, and versatile cytoskeletons as hallmarks of eukaryotic life. Moreover, we identify microfluidics and DNA nanotechnology as two technologies that can integrate these functional modules into sophisticated multifunctional synthetic cells.

## Highlights

Bottom-up synthetic biology thrives in reverse-engineering a particular biological function using a minimal set of molecular components, like purified proteins.

Recently, precision technologies, like microfluidics, have been used to recombine functional modules towards multifunctional synthetic cells. Synthetic biology can capitalize on a variety of pre-existing on-chip functions, which greatly increases the scope for complexity in the field.

Advances in DNA nanotechnology gave rise to a diverse range of fully synthetic functional modules, like DNA-based ion channels or motors, which can replace some protein-based parts.

Noteworthy progress has been made in achieving large and stable compartments, organelle-like multicompartment systems, and sophisticated cytoskeletal structures.

## Engineering Eukaryotic Cells from the Bottom up

Every cell found on Earth today originates from a pre-existing cell. In principle, however, the emergence of life on Earth shows that it is possible to engineer life from its molecular constituents. This, in turn, leads to a philosophical question: Can the transition between nonliving and living matter be achieved in a laboratory? The audacious vision of building an entire cell from scratch has recently been transferred into the realm of the possible – well aware that, if successful, a **synthetic cell** (see Glossary) would revolutionize our understanding of the origin and the future of life. The quest for a synthetic **eukaryote** is in the first instance a merely curiosity-driven scientific endeavor with the overarching aim to understand life in greater detail. It may offer means to probe theories on the evolutionary origin of the eukaryotic cell, which remains one of the greatest mysteries in evolutionary biology [1]. Moreover, synthetic eukaryotic cells will also be indispensable for visionary applications, especially when it comes to synthetic phagocytotic systems, programmable drug carriers, or replacements for deficient cells. Towards this end, the young and dynamic field of **bottom-up synthetic biology** has been successful at dissecting cellular phenomena to study them in isolation. It has produced **cellular functional modules**, such as energy modules or gene expression modules, with reduced complexity. Several recent reviews portray these developments 2, 3, 4, 5. A comparison of top-down and bottom-up approaches to synthetic biology is presented in Box 1.

Box 1 **Bottom-up versus Top-down Synthetic Biology**

Synthetic biology aims to construct artificial living systems with programmable functionality. In the literature, the term synthetic cell has been used broadly, sometimes inconsistently, describing systems from a cell-sized compartment to a genetically engineered living cell. This is largely due to the existence of two different but complementary approaches to synthetic biology.

The top-down approach derives a synthetic cell from a biological cell by manipulating, for example, its genes and protein content. This type of synthetic cell is typically living and still closely related to its biological ancestor. A well-known example for top-down synthetic biology is the minimal genome projects, which aim to identify the essential genes of a given species. Such genomes have successfully been booted inside living cells [58].

The bottom-up approach, on the contrary, starts with nonliving matter. Its most basic synthetic cell is merely a cell-sized compartment. Cell-like functionality is derived by reconstituting functional modules, made from natural or artificial molecular building blocks. The complexity of the synthetic cell is increased step-by-step, by including more and more components. This strategy has successfully been used to reveal the biological role of individual proteins and may one day result in the first truly synthetic living cell without biological ancestry.

Alt-text: Box 1

**Figure 1:**
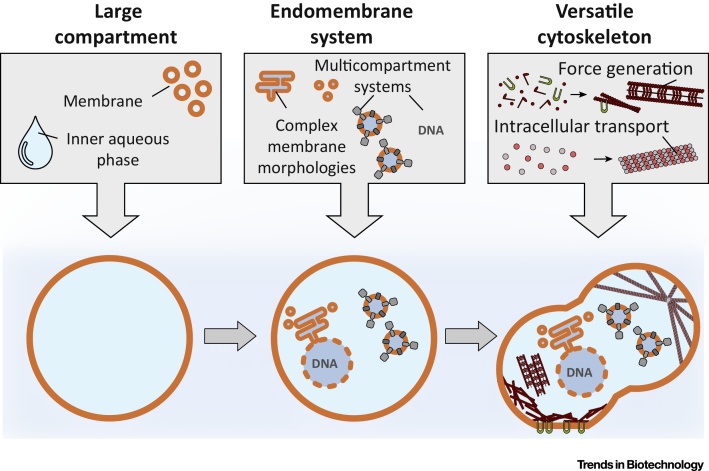
Key Figure: Schematic Illustration of a Sequential Assembly Strategy for Multifunctional Synthetic Eukaryotes

Realizing the vision of a fully functional synthetic cell, however, will require strategies to recombine the mosaic of functional modules inside cell-like compartments. In this review, we explicitly highlight approaches that, in principle, allow for the stepwise integration of functional modules towards a multifunctional system (Figure 1, Key Figure). Distinguishing such efforts from the certainly not less exciting work towards minimalistic **protocells** becomes important as the library of functional modules is growing. We propose the integrability as the key criterion according to which synthetic cellular modules should be rated, which will help to compare and contrast achievements in the field. We further propose a classification of synthetic cells according to the pre-existing divide between prokaryotic and eukaryotic cells. The characteristics of a synthetic eukaryote are prescribed by the distinguishing features of nature’s own archetype: (i) large cell size compared to prokaryotes; (ii) complex **endomembrane system** with multiple organelles; and (iii) distinctive multifunctional cytoskeleton. These three features define the sections of this review as illustrated in Figure 1.

## Large Compartment Size

Typically tens of microns in size, eukaryotes are on average larger and enclose 100–10 000 times greater volumes compared to prokaryotic cells [6]. The physical dimensions have a profound impact on cellular processes affecting, among other things, the surface-to-volume ratio and the diffusion time of reagents. A cell-sized compartment as a physical container of cellular function is thus the most basic unit of a synthetic eukaryote. Such compartments have been made from a diverse group of amphiphilic vesicle forming molecules, including coacervates [7], polymer- or lipid-based water-in-oil droplets 8, 9 and water-in-water **polymersomes** [10]. The most frequently chosen compartment type are **giant unilamellar vesicles (GUVs)**. Their key advantage lies in the use of lipids, which, as the building blocks of natural cell membranes, allow for the reconstitution of many cellular components without loss of functionality. GUVs are commonly produced by electroformation [11], or alternatively by gentle hydration 12, 13, microfluidic jetting [14], and solvent evaporation methods [15]. Unfortunately, these techniques often require specific buffers and lipid compositions that may be incompatible with specific cellular modules. The encapsulation efficiency is typically low, meaning that the molecularly crowded environment of a living cell cannot be mimicked efficiently.

**Figure 2:**
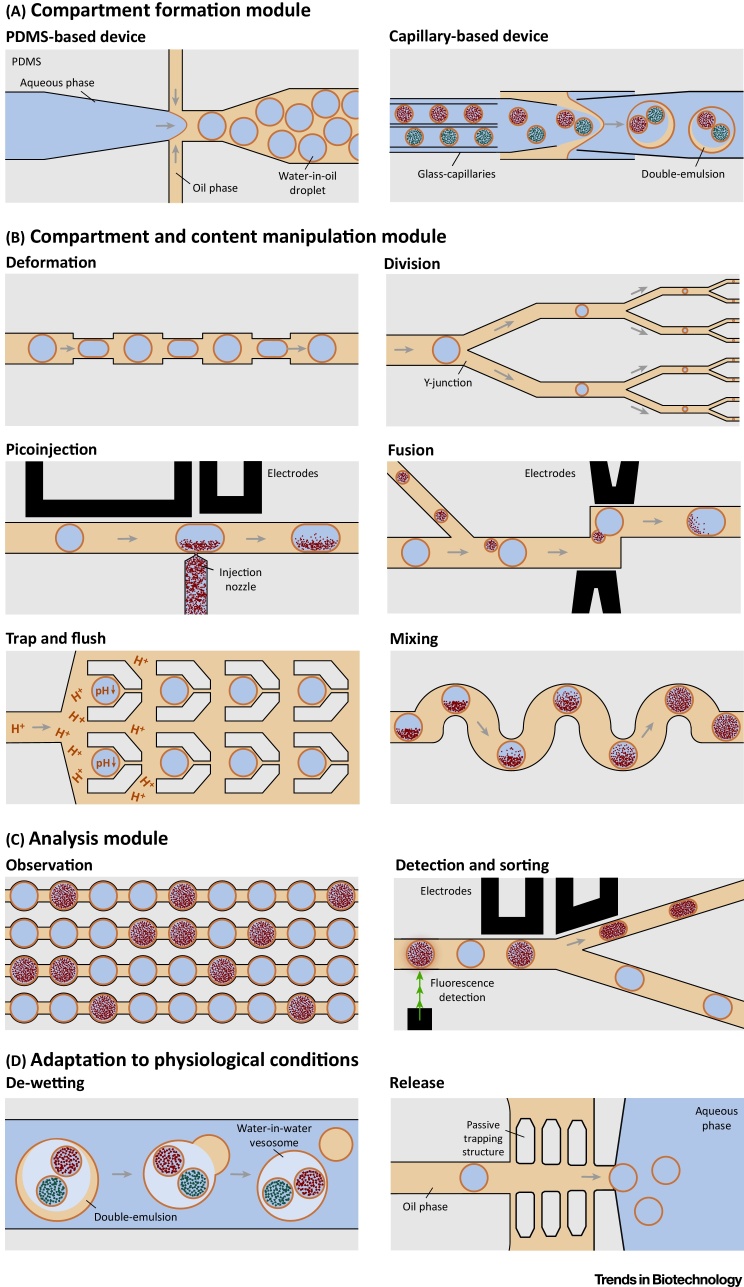
Schematic Representation of Droplet-based Microfluidic Units Categorized into Four Major Functional Modules for Synthetic Biology Applications For a Figure360 author presentation of Figure 2, see the figure legend at https://doi.org/10.1016/j.tibtech.2018.03.008 Figure360: an author presentation of Figure 2Figure 2 (A) Compartment formation module: flow-focusing junction of a **polydimethylsiloxane (PDMS)**-based device for the formation of water-in-oil droplets and capillary-based device for the formation of single- and multicompartmented double emulsions in water. (B) Compartment and content manipulation module: deformation units are used to achieve arbitrary compartment shapes due to a change in flow regime, facilitating content mixing and investigation of mechanical properties. Division units can be used for mechanical splitting of the compartment and its content (either symmetrically or asymmetrically) at a microfluidic Y junction to mimic cell division. Picoinjection units use electroporation to transfect the synthetic cells with the desired biocomponents. Picoinjection cycles allow for the sequential addition of multiple components. Fusion units are used to achieve compartment fusion, for example, via an electric field. The process mimics vesicle-based cellular transport and can be used for the transfection of large substrates. Picoinjection and fusion units can also mimic cell growth. The trap and flush unit contains cavities in a flow chamber to trap compartments for changing environmental conditions like pH or for supplying reagents externally. The mixing unit achieves fast homogeneous distribution of reagents inside the compartment. (C) Analysis module: the dropspot chamber unit is used for time-lapse observation of multiple compartments. In the fluorescence-activated sorting unit, an electric field electric field is triggered by the fluorescence readout of the compartment. The compartments will be deflected into the narrow channel at the Y junction due to the dielectrophoretic force, otherwise the droplets flow into wider channel due to the lower hydraulic resistance. This unit can be used to mimic cell death and evolution. (D) Adaptation to physiological conditions: de-wetting and release units allow for oil and polymer shell removal to transfer the assembled compartments to physiological conditions.

More recently, **microfluidics** has been used to produce GUVs on a chip by de-wetting of double-emulsion droplets (Figure 2) 16, 17. Even mechanical on-chip division has been achieved [18]. The advantage of microfluidic methods is that GUVs can be produced at kilohertz rates with unprecedented control over the uniform compartment size. Moreover, the choice of membrane composition and buffer conditions is more flexible compared to the standard methods. Functional modules or reagents can be directly encapsulated in the aqueous inlet during the formation process of the GUVs. However after formation, the manipulability remains limited as GUVs are chemically and mechanically unstable [19]. This means that it is difficult to co-reconstitute reagents sequentially if they cannot be mixed together in one pot during compartment formation. Recently, Weiss and colleagues demonstrated a high-throughput, droplet-based microfluidic approach to generate stable, defined-size vesicles termed droplet-stabilized GUVs (dsGUVs) [20]. dsGUVs can be loaded sequentially with multiple different components or functional modules by means of **picoinjection** microfluidics [21] before being released from the oil phase and from the polymer shell into a physiological environment. Developments like these leave no doubt that microfluidics will increase the scope for complexity in the field of bottom-up synthetic biology 22, 23. We highlight the applicability of such microfluidic units throughout this review (Box 2 and Figure 2).

Box 2 **Microfluidic Modules for Synthetic Biology**

Microfluidic technologies have reached maturity, offering a high level of reproducibility, automation, and manipulability. On-chip functions have been developed independently, but their transfer into the field of synthetic biology will help inspire and kick start completely new assembly and assessment strategies for synthetic cells.

Synthetic biology can capitalize on diverse pre-existing microfluidic units. In the context of bottom-up synthetic biology, these units can be categorized into four major functional modules as illustrated in Figure 2. (i) The compartment formation module that encompasses microfluidic units for the formation of cell-sized single- and multicompartment systems. (ii) The compartment and content manipulation module which can be used to increase the complexity of synthetic cells in a sequential manner. Microfluidic units in this module can achieve growth and division, mimicking a simple cell cycle. (iii) The analysis module with units designed to detect and to monitor the content of synthetic cells systematically. Sorting units may be used to mimic cell death and evolution of heterogeneous populations of synthetic cells. (iv) The adaptation module that contains units to adapt the environment of the developed cell-like compartments to physiological conditions, for instance by de-wetting double emulsion templates or release into an aqueous environment.

Alt-text: Box 2

At this point it seems likely that a synthetic eukaryote will be enclosed by a lipid membrane, as this approach allows for the straight-forward inclusion of natural building blocks. Moreover, this strategy will allow for the formation of a complex endomembrane system, the next step towards architectural mimicry of a eukaryotic cell.

## Endomembrane System with Organelles

Eukaryotic life is characterized by the coexistence of various internal membrane structures surrounding the nucleus and other organelles. Organized hierarchically, dedicated compartments take control of crucial functions including, but not limited to, nucleic acid production, material storage, and energy production. In this section, we first focus on processes to obtain synthetic multicompartment cells. We then showcase achievements towards synthetic cell nuclei and mitochondria as two key organelles and finally discuss strategies to obtain complex membrane morphologies beyond the archetypal spherical shape.

### Multicompartment Vesicles

Synthetic cells with multiple functional compartments represent a crucial first step towards the structural mimicry of eukaryotes. They offer a route towards higher-order functions by uncoupling enzymatic reactions, concentrating reagents, and separating incompatible cellular modules. Synthetic multicompartment vesicles, also referred to as multivesicular vesicles, nested vesicles, or **vesosomes**, can emerge from spontaneous or induced [24] endobudding of GUVs. They have also been formed by mimicking the natural process of endosymbiosis [25]. However, these methods cannot provide the desired control over number, cargo and membrane composition of the inner compartments.

Overcoming these limitations, Deng and colleagues achieved precise control over compartment number and content in a high-throughput manner using a capillary-based microfluidics as illustrated in Figure 2A [26]. They also demonstrated the size-selective transfer of fluorescent dyes between inner and outer compartment by incorporating protein pores [26] – a first step towards regulating chemical and electrical communication between the compartments. However, the major drawback of this current technology is that the compartments cannot be modified or manipulated after their formation. This could potentially be addressed by inserting preformed internal compartments via microfluidic picoinjection unit as illustrated in Figure 2, or by adapting the picoinjection technology to water-in-water systems.

**Figure 3:**
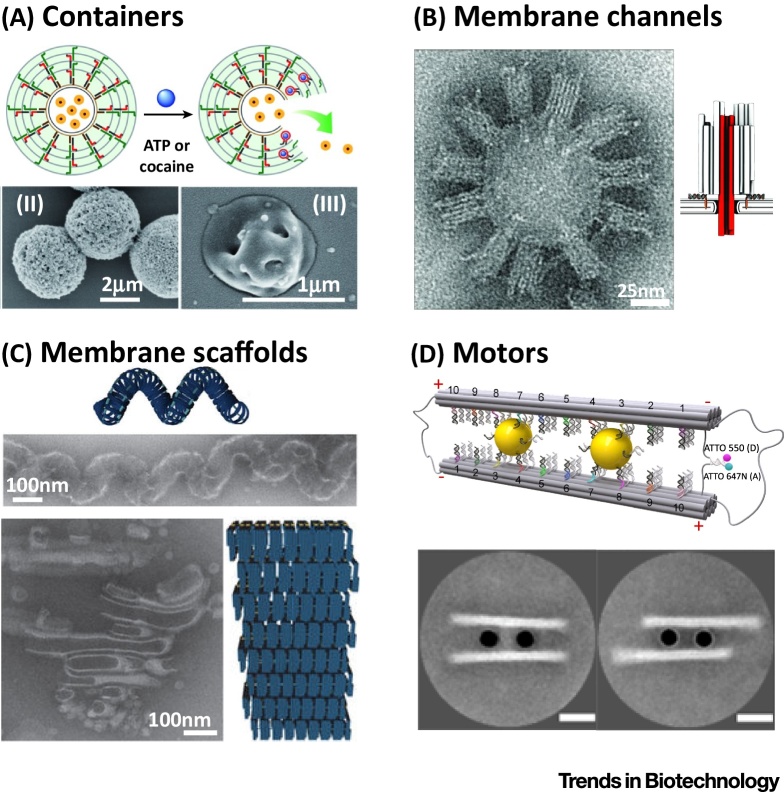
Schematic representation and Electron Microscopy Micrographs of Several DNA-based Modules for Synthetic Cells. (A) Containers for stimuli-responsive cargo release, here DNA–acrylamide hydrogel microcapsules. Reprinted, with permission, from [30]. (B) Membrane-spanning DNA-based mimics of ion channels with customizable sizes and properties. Reprinted, with permission, from [42]. (C) Complex membrane architectures scaffolded with DNA. Reprinted, with permission, from [34]. (D) DNA-based motors and walkers fuelled by strand-displacement reactions. Reprinted, with permission, from [48].

Multicompartment systems have recently also been introduced into living cells, where they perform enzymatic cascade reactions [27]. Such developments open up a route towards smart synthetic cells capable of *in vivo* diagnostics and algorithmic release of hierarchically organized compounds. In the context of synthetic biology, internally structured vesicles are key to reverse-engineer the morphology and functionality of eukaryotic cells. They can serve as model systems to mimic endosymbiosis or molecular and supramolecular processes that occur in living cells. Yet, this ultimately requires strategies to organize the internal compartments. Here, the programmability of DNA provides interesting opportunities: single-stranded DNA has been covalently modified with hydrophobic moieties that self-assemble into lipid membranes. This way, lipid compartments tagged with cDNA sequences have been positioned in a programmable and reversible manner [28]. More generally, synthetic biology can capitalize on a pre-existing toolbox of functional units that were developed in the field of **DNA nanotechnology** over the past decades (Table 1, Figure 3, and Box 3). Progress towards synthetic cell nuclei and mitochondria as two key compartments of eukaryotes will be discussed in the following sections.

**Table 1:** DNA Nanotechnology-based Mimics of Cellular Components for Synthetic Cells

Cellular component	DNA mimic and its function	In/on GUVs?	Refs
Exosomes, transport vesicles	DNA capsules for stimuli-controlled release of cargo	No	[30]
Epsin, clathrin	Curvature-imposing DNA structures for membrane bending	Yes	31, 32, 33
Tubulation of organelles	DNA-based scaffolds for complex reconfigurable membrane architectures	No	34, 35, 36
Cytoskeletal proteins	Membrane-bound DNA-based lattices for compartment stabilization	Yes	[37]
Ribosomes	DNA-based assemblers for synthesis of polymers with programmed sequence	No	[38]
Antibodies	DNA aptamers for molecular recognition	Yes	39, 40
Ion channels, porins	DNA-based membrane pores for electrical and chemical communication between compartments	Yes	41, 42, 43
Scramblases	Membrane-spanning DNA constructs for transport of lipids between bilayer sheets	Yes	[44]
SNARE proteins	Membrane-anchored DNA to mediate compartment fusion	Yes	[45]
E-cadherins	DNA-based compartment linkers for reversible and programmable assembly of multicompartment systems	Yes	[28]
Actomyosin, microtubules, kinesin	DNA-based molecular walkers for programmable cargo transport	No	46, 47, 48
Light-harvesting complexes	DNA origami platforms for rationally designed antenna structures	No	[49]

Box 3 **DNA Nanotechnology for Synthetic Biology**

DNA nanotechnology uses DNA, beyond its purpose in nature, as a construction material to build arbitrary objects at the nanometer scale. It is based on the realization that the self-assembly of hundreds of short DNA strands can be programmed by exploiting the universal base-pairing rules of DNA [29]. Over the past decade, rapid prototyping via computer-aided design and the rich toolbox for the site-selective functionalization led to a myriad of folded DNA architectures (**DNA origami** [87]), nanodevices, and sensors. In the context of synthetic biology, it can be used to either assemble pre-existing components in a programmable manner or to build artificial components that are difficult to obtain otherwise. In recent years, several DNA-nanotechnology-based modules have been developed for synthetic cells as listed in Table 1 and highlighted in Figure 3.

Alt-text: Box 3

### Cell Nucleus

Of all membrane-enclosed compartments, the nucleus is the defining part of the eukaryotic cell – giving the domain of life its name. It stores and organizes the genetic code, while making its information accessible for the production of proteins. Thus, the incorporation of a cell nucleus or its mimic is of special interest for synthetic eukaryotes.

The simplest strategy is to encapsulate the functional nucleus of a living cell into a synthetic compartment. While the resulting hybrid may not be fully synthetic, this experiment led to an important insight: it revealed that the size of the mitotic spindle could be influenced by changing the compartment volume alone 50, 51. However, control over the function of the cell nucleus is still limited due to the complexity of the eukaryotic genome. Therefore, Deng and colleagues recently presented the first simple artificial mimic of a cell nucleus within a lipid-based synthetic cell: by means of capillary-based microfluidics (Figure 2), they embedded a liposome containing an *in vitro* transcription mix into larger compartments [26]. In this system proteins were produced directly in the internal DNA-containing compartment. The highly challenging next step is to transfer RNA across the inner compartment membrane to achieve the spatial separation of transcription and translation which characterizes eukaryotes. This requires the use of a membrane pore that is large enough to allow for the passage of RNA, but impermeable to all precursors needed for the transcription process. In nature, the selective passage of RNA is achieved by the nuclear pore complex, which, until now, cannot be reconstituted into a synthetic system in its functional form.

Large protein nanopores that remain open for extended periods of time are rare and often difficult to purify. Here, an artificial pore may be easier to obtain: a DNA origami nanopore that matches the electrical diameter of the nuclear pore complex has already been demonstrated [41], and single-stranded DNA has been translocated through DNA pores under an applied electrical field [42] (Table 1). Yet, current examples of DNA-based pores lack the sophisticated selectivity of the nuclear pore complex. Positioning peptides from the nuclear pore complex on a DNA origami scaffold as shown recently 52, 53 could be a promising approach. Circumventing the need for a pore, microfluidic picoinjection, or fusion technologies (Figure 2) could achieve the transfer of preformed mRNA into the synthetic compartment. Alternatively, the membrane of the synthetic nucleus could be made from a more porous material instead of lipids, like stimuli-responsive DNA-based capsules (Table 1) [30]. Also nucleocapsids could serve as mimics of a cell nucleus. Such capsids, capable of evolution, have recently been made from synthetic proteins [54]. Recently, Krinsky and colleagues demonstrated synthetic DNA-containing cells made of a single compartment capable of synthesizing therapeutic proteins inside tumors [55].

Still, *in vitro* expression of one or a few proteins cannot compete with the complexity of a eukaryotic genome, where thousands of different proteins are produced in a genetically regulated manner. Using a top-down approach, minimal eukaryotic genomes have been designed and partially synthesized [56]. Smaller minimal prokaryotic genomes have even been booted in living cells 57, 58, but never in synthetic cells. While it is possible to create both, partially functional synthetic cell nuclei and fully functional semisynthetic genomes, putting the two together and booting a full genome inside a synthetic cell remains an unachieved and still distant goal.

### Mitochondria

Energy generation is the key process to sustain life in an out-of-equilibrium state. In cellular systems, protons are pumped across a membrane to establish a proton gradient, which is then often transformed into the chemical energy currency **ATP** [59]. Maintenance and replication of complex internal membrane structures and large genomes requires an increased amount of ATP. It has been proposed that energy limitations in prokaryotes may constrain their complexity [60]. Under this scenario, the internalization of the energy generation, hence the acquisition of mitochondria, was the key step en route to the more complex eukaryotic cell plan. Autonomous synthetic eukaryotes will thus need a form of artificial mitochondria.

A minimal system for ATP production requires an enzyme that can establish a proton gradient in combination with ATP synthase or a synthetic analog, which dissipates the energy from the proton gradient to produce ATP. The light-driven proton pump bacteriorhodopsin and the enzyme ATP synthase have successfully been co-reconstituted into small unilamellar vesicles [61] and polymersomes [62]. Instead of light, it is also possible to use the energy from the reduction of oxygen for ATP production by replacing bacteriorhodopsin with ubiquinol bo3 oxidase in small unilamellar vesicles 63, 64 and polymersomes [65]. Their co-reconstitution has also been achieved in cell-sized GUVs [66], where ATP synthase has been shown to induce nonequilibrium membrane fluctuation [67]. The next step is the encapsulation of such artificial mitochondria into a bigger compartment to mimic the architecture of a eukaryotic cell. Here, the microfluidic encapsulation or picoinjection of small vesicles into a large compartment or vesosomes (Figure 2) might be a promising strategy.

Due to the unfavorable surface area to volume ratio, energy production across the external membrane is unlikely to sustain vital energy-intensive processes in eukaryotes – synthetic or natural. Natural mitochondria have a complex and folded inner membrane architecture with so-called cristae to provide additional space for the enzymes of the electron transport chain. Khalifat and colleagues observed the appearance of cristae-like invaginations in the presence of a local pH gradient, proposing a model to explain the membrane morphology of mitochondria [68]. Yet, not only mitochondria are characterized by their complex membrane architectures as described in the next section.

### Complex Membrane Morphologies

Endocytic pits, filopodia, apoptotic blebs, or the endoplasmic reticulum (ER) are examples of dynamic function-related membrane structures in eukaryotes. In synthetic compartments, however, the energetically favorable spherical shape remains predominant. Cells achieve membrane deformations inter alia via specialized membrane-inserting proteins. Reconstitution of such membrane-bending proteins, like epsin, into synthetic compartments revealed the concentrations required for membrane bending [69] and the mechanisms by which intrinsically disordered proteins cause membrane tubulation [70]. Similarly, amphiphatic DNA-based constructs have been used as synthetic analogs to drive membrane tubulation [31] (Table 1). By inserting clathrin into GUVs, it has been shown that the balance between membrane elasticity and polymerization energy sets the shape of clathrin pits involved in endocytosis [71]. DNA nanotechnology, however, is currently the only strategy by which custom-designed, arbitrary, and reconfigurable lipid architectures can be obtained in a reproducible manner: a DNA template defines the shape of the lipid compartment and triggers the formation of a lipid bilayer on its surface via hydrophobic tags 35, 36. Using this method, Zhang and colleagues recently demonstrated helical lipid tubes or ER-like structures that can be remodeled dynamically by reconfiguring the DNA template [34] – again highlighting the versatile applicability of DNA nanotechnology for synthetic cells. The encapsulation of such lipid architectures into larger compartments should be straightforward, for instance by means of microfluidic technologies as presented in Figure 2. Spherical and tubular lipid architectures have been released from their DNA shell by means of DNA-digesting enzymes 35, 36. More complex shapes, however, are energetically unstable and have to be sustained via an internal or external cytoskeleton mimic. Approaches to obtain such a cytoskeleton are discussed in the next section. Nevertheless, it will still take time before the dynamic membrane architecture of a eukaryotic cell can be modeled and faithfully copied in a synthetic system. Therefore, it appears likely that the first synthetic eukaryote will predominantly rely on a spherical multicompartment architecture.

## Versatile Cytoskeleton

As a network of interlinking filaments extended throughout the cytoplasm, cytoskeletal elements share important roles in prokaryotes and eukaryotes, such as chromosome segregation and cytokinesis [72]. Cytoskeleton associated motor proteins for actin-based force generation and intracellular transport, however, are unique to eukaryotes and will be subject of this section.

### Actomyosin-Based Force Generation

Filamentous actin (F-actin) polymerizes into polarized filaments, which favor growth at one end and shrinkage at the other. This ATP-driven process allows for rapid actomyosin cytoskeletal changes that generate forces for processes like motility or endocytosis. Minimal actin cytoskeleton systems have been reconstituted mainly in bulk. These works provided important insights related to the biophysical process of actin cytoskeleton formation and its functions. Imposing confinement, contractile actomyosin rings have been demonstrated in water-in-oil droplets in the presence of bundling factors [73]. Such contractile rings initiate the division of many eukaryotic cells. In the presence of actin and a minimal set of actin-binding proteins, Liu and colleagues observed filopodium-like protrusions [74]. This means that the membrane alone can facilitate the transition of a branched actin network to a parallel array of actin filaments. The formation of such protrusions can be considered as the first step towards reconstituting protrusion-based cellular motility. In many cases, motility requires cells to adhere and to interact with the extracellular environment. Towards this end, reconstitution of surface receptors interlinking the extracellular matrix and the internal cytoskeleton in synthetic cells is a necessary step. To achieve substrate-specific adherence, functional integrins have been reconstituted into GUVs by means of microfluidic picoinjection [20] or by reconstitution during GUV electroformation [75]. The fact that no enrichment of integrins was observed in the adhesion patches [75] indicates the important contribution of the cytoskeleton to the formation of focal adhesions. This is underlined by the observations of Murrell and colleagues who reported cell-like spreading of a GUV with an actin cortex alone [76]. Obviously, the next key step would be to find a minimal set of components – natural or artificial – linking integrins to the actin cortex. The joint reconstitution of these components is unlikely to be achievable in a one-step reaction. A potential strategy could be to assemble integrin-GUVs first and to supply the linkers and actin in a subsequent step, for instance via microfluidic picoinjection or fusion units (Figure 2).

The last step in the protrusion-based migration process is the myosin-based contraction of the actin network, combined with the detachment of the rear focal adhesions. Synthetic cells served as model systems to study how myosin builds tension in actin networks. Experiments reconstituting actin on the inside or on the outside of GUVs showed that cortex connectivity and membrane attachment govern the contraction outcome: cortices contract inward when weakly attached, whereas they contract towards the membrane when strongly attached [77]. Cortical flows and spontaneous symmetry breaking reminiscent of the initial polarization in embryo development was observed in actomyosin-loaded GUVs 78, 79 and in water-in-oil droplets [80]. While none of the systems described above show movement, it now seems to be an achievable goal. If the motility can be self-sustained via the internal production of ATP, as described in Mitochondria section, this would be a remarkable milestone towards autonomous synthetic cells. Progress in this direction will almost certainly rely on strategies to transfer the functional components step-by-step into a preformed compartment as demonstrated via microfluidics.

### Microtubules and Intracellular Transport

Together with actin and intermediate filaments, microtubules form the eukaryotic cytoskeleton. They are capable of generating force via directional growth and serve as tracks for motor proteins, which carry organelles and other components to their target location. Compelling evidence exists that microtubules and molecular motors of the dynamin and kinesin families are involved in the tubulation of organelles, thereby establishing the architecture of eukaryotic cells.

It has been shown that a minimal system consisting of GUVs and externally supplied microtubules and kinesin can produce membrane networks in the presence of ATP and GTP − without the aid of other proteins [81]. Increasing the level of complexity one step further, a basic minimal endocytosis module was reconstituted: the addition of dynamin caused membrane fission and vesicle release [82]. It should be noted, however, that all reagents were not encapsulated within the GUV but added externally. Their reconstitution inside a lipid compartment should be straightforward using microfluidic encapsulation or picoinjection (Figure 2). This could be a strategy for the autonomous creation of a multicompartment system. As a first step, tubulin has already been polymerized inside microfluidic droplets 20, 83. Remarkably, Sanchez and colleagues demonstrated spontaneous motion of droplets loaded with microtubules and kinesin, likely caused by cytoplasmic streaming [83]. Juniper and colleagues presented a robust microfluidic system to study motor protein and microtubule self-organization in polymer-stabilized droplets of well-defined size. An important outcome of this study was that confinement can impose a novel pathway for microtubule aster formation via the constriction of an initially spherical motor-microtubule network. The observed mechanism illustrates the close relationship between confinement, network contraction, and aster formation [84]. With the successful reconstitution of actin contractile rings [73] and microtubule asters, the controlled division of synthetic cells may be an achievable goal. It will be necessary to find suitable linkers between the cytoskeletal components and to combine the system with an ATP-generating module.

Deviating from natural systems, DNA-based motors that walk along a track are the most promising candidates to recapitulate intracellular transport in a purely synthetic manner. In these systems, movement is fuelled by the hybridization and subsequent hydrolysis of DNA base pairs between the DNA-based motor and its track. Long-range transport with rates up to 2 μm/min [85], as well as programmable navigation through branched track networks [86] and cargo sorting [46], have been demonstrated. Recently, regulatory inputs in the form of DNA strands were provided to the motor sequentially by means of microfluidics increasing its performance [47]. All these systems have not yet been encapsulated into lipid compartments, although this step should be well within reach.

Recently, a DNA lattice has been absorbed onto the inner compartment membrane of a GUV [37]. So far, the only function of this synthetic cytoskeleton is to stabilize the synthetic cell. In principle, however, it could serve as a track for DNA-based motors and provide anchoring points or linkers for other modules of synthetic cells.

## Concluding Remarks

In the past two decades, bottom-up synthetic biology has successfully reconstituted various phenomena occurring in living systems. Emphasis is now being put on strategies to integrate functional modules into multifunctional systems. Until recently, the lack of technologies for the sequential assembly and manipulation of synthetic cells was limiting the progress. This hurdle has largely been overcome thanks the introduction of precision technologies, like microfluidics, into the field. Previously developed functional modules can now, in principle, be assembled in a step-by-step manner with unprecedented control. These functional modules, however, may lose their activity when recombined due to undesired chemical or physical interactions or incompatible buffer conditions (see Outstanding Questions). Here, mimicking the architecture of eukaryotes with their multiple compartments and segregated reaction pathways holds the key to success. Engineering sophisticated membrane pores will then be necessary to allow for communication and selective exchange of reagents.

Some cellular modules, however, will remain difficult to reconstitute. In this case, artificial mimics may be an adequate solution. Especially, the field of DNA nanotechnology offers new opportunities: as highlighted in this review, DNA-based constructs have successfully been used to program the assembly of cellular components and to achieve functionality that is normally provided by proteins. Additionally, the integration of synthetic designer proteins and computational tools for their structure prediction will increase the versatility of synthetic cells.

Moving away from naturally occurring building blocks is conceptually interesting and offers a route towards truly synthetic systems, which has profound implications for the definition of life in itself. In some cases, however, it will remain difficult to match the efficiency and sophistication of engineering solutions offered by nature. Therefore, a combination approach using both natural and synthetic building blocks will likely be the best strategy. It will also be enriching to recombine bottom-up and top-down approaches to synthetic biology.

In the coming years, the integration of evolution into the conceptual toolbox of bottom-up synthetic biology will lead to exciting developments. So far, there is no study that has convincingly demonstrated open-ended evolution in a synthetic system. Additionally, a long-term goal is to integrate synthetic cells into a compartment hierarchy analogous to the organelle – cell – tissue – organ structure of multicellular systems. Here, it will be powerful to combine algorithmic self-assembly facilitated by DNA nanotechnology with microfluidic bioprinting of synthetic cells. After all, it was ultimately the emergence of multicellularity that gave rise to the mesmerizing diversity of life on Earth. We may thus be looking forward to witness future developments in this emerging field, when scientific curiosity continues to follow the intrinsic motivation of human endeavor – to discover the secret of life. Just like life will keep converging towards the best solutions, so will, no doubt, the myriad of approaches to synthetic biology.

Outstanding Questions What technologies, apart from microfluidics, allow for the sequential assembly of synthetic cells?

Is there a universal ‘synthetic intracellular buffer’ that can be used for the assembly of all types of functional modules? Alternatively, can compartmentalization achieve the recombination of functional modules without loss of function?

Which functional modules could or should be replaced with fully synthetic building blocks assembled, for example, using DNA nanotechnology? Can such hybrid systems perform new functions that are not found in nature?

How will it be possible interface synthetic and natural cells and to create symbiotic cell communities? How can such systems be harnessed for applications, for example, in immunology or wound healing?

How can synthetic cells be integrated into a compartment hierarchy analogous to the organelle – cell – tissue – organ structure of multicellular systems? Will it be possible to create tissues and eventually organs from synthetic cells?

How can open-ended evolution be achieved in a synthetic cellular system?

Will it be possible to achieve a fully synthetic cell which departs from nature’s own blueprint of cellular life and uses only artificial materials? What are the implications for the definition of cellular life?

How can sophisticated hybrid cells be achieved, recombining building blocks from natural and synthetic cells? To give an example, will it be possible to boot a genome inside a synthetic compartment?

Which ethical principles should be established for artificial forms of cellular life? At what point do they have to be applied?

## Glossary

**Adenosintriphosphat (ATP)**: organic chemical that mediates energy transfer in metabolic processes in all cells.
**Bottom-up synthetic biology**: emerging research field that aims to construct biological functions and ultimately cells from natural, modified or synthetic biomolecules. Complementary to the top-down approach, which modifies pre-existing cells.
**Cellular functional module**: minimal system that can perform a certain cellular function, for example, energy module for the production of ATP.
**DNA nanotechnology**: design and construction of artificial nucleic-acid-based nanostructures, exploiting the base pairing properties of DNA beyond their role in genetics allowing for the assembly of programmable functional materials.
**DNA origami**: common technique in DNA nanotechnology, involves folding single-stranded genomic phage DNA (scaffold) by attachment of hundreds of short synthesized DNA oligonucleotides (staples), achieves arbitrary fully addressable 2D and 3D nanostructures.
**Endomembrane system**: entirety of all internal membranes of a eukaryotic cell, including organelles.
**Eukaryotes**: organisms belonging to the domain Eukaryota, which are distinguished from prokaryotes due to their larger cell size, membrane-bound organelles, and versatile cytoskeleton.
**Giant unilamellar vesicle (GUV)**: spherical micron-scale capsule enclosed by a lipid bilayer, used as the most common model membrane system for synthetic cells.
**Microfluidics**: technology to enable handling, processing, and manipulation of fluids of picoliter volumes via PDMS-based microchannels or glass capillaries. It permits the integration of multiple laboratory functions into one microfabricated chip, requires minimal manual user intervention and sample consumption, and allows for enhanced data analysis speed and precision.
**Picoinjection**: microfluidic electroporation-based method to inject picoliter volumes of aqueous solutions into preformed water-in-oil droplets.
**Polymersomes**: water-in-water polymer-based vesicles, sometimes used as cell like compartments instead of lipid-based membrane systems due to higher mechanical stability.
**Polydimethylsiloxane (PDMS)**: silicon-based viscoelastic organic polymer, widely used in microfluidics and beyond due to its biocopatibility.
**Protocell**: self-replicating minimal lipid-based compartments proposed as a stepping stone to the origins of life. Most basic but not yet achieved form of a functional synthetic cell.
**Synthetic cell**: cell of non-natural origin that has been obtained either by modifying a pre-existing cell (top-down assembly) or by assembling compartments that mimic one or more properties of a living cell (bottom-up assembly). Sometimes also referred to artificial cell, protocell, cell-like compartment or synthetic cellular robot.
**Vesosome**: a vesicular compartment that contains internal subcompartments, also referred to as multicompartment or multivesicular vesicles, nested vesicles.
